# The StartReact Effect on Self-Initiated Movements

**DOI:** 10.1155/2013/471792

**Published:** 2013-09-11

**Authors:** J. M. Castellote, M. E. L. Van den Berg, J. Valls-Solé

**Affiliations:** ^1^Physical Medicine and Rehabilitation Department, Faculty of Medicine, Universidad Complutense de Madrid, C/Ciudad Universitaria S/N, 28040 Madrid, Spain; ^2^National School on Occupational Medicine, Instituto de Salud Carlos III, C/Sinesio Delgado 4, 28029 Madrid, Spain; ^3^Division of Rehabilitation, Aged Care and Allied Health, Repatriation General Hospital, Flinders University, Daw Park, SA 5043, Australia; ^4^EMG Unit, Neurology Department, Hospital Clínic, Centro de Investigación Biomédica en Red sobre Enfermedades Neurodegenerativas (CIBERNED), Institut d'Investigacio Biomedica August Pi i Sunyer (IDIBAPS), Facultad de Medicina, Universitat de Barcelona, Calle Villarroel 170, 08036 Barcelona, Spain

## Abstract

Preparation of the motor system for movement execution involves an increase in excitability of motor pathways. In a reaction time task paradigm, a startling auditory stimulus (SAS) delivered together with the imperative signal (IS) shortens reaction time significantly. In self-generated tasks we considered that an appropriately timed SAS would have similar effects. Eight subjects performed a ballistic wrist extension in two blocks: reaction, in which they responded to a visual IS, and action, in which they moved when they wished within a predetermined time window. In 20–25% of the trials, a SAS was applied. We recorded electromyographic activity of wrist extension and wrist movement kinematic variables. No effects of SAS were observed in action trials when movement was performed before or long after SAS application. However, a cluster of action trials was observed within 200 ms after SAS. These trials showed larger EMG bursts, shorter movement time, shorter time to peak velocity, and higher peak velocity than other action trials (*P* < 0.001 for all), with no difference from Reaction trials containing SAS. The results show that SAS influences the execution of self-generated human actions as it does with preprogrammed reaction time tasks during the assumed building up of preparatory activity before execution of the willed motor action.

## 1. Introduction

Movement execution requires previous preparation of the motor system at various levels. In reaction time tasks, several authors have gathered evidence for an enhancement of motor cortex excitability preceding onset of electromyographic (EMG) activity in the agonist muscle [1–5]. At the same time there is an increase in excitability in subcortical motor structures, as shown by the premovement increase in the size of the H reflex [6] and the enhancement of the startle response [7]. In self-generated movements, the decision to start the movement seems to be an act of free will, performed without an apparent external cue. Nevertheless, there are changes in brain activity that precede the generation of muscle EMG activity and consequent contraction. These can be seen as a slowly rising negativity beginning from 1.5 to 2 s before onset of EMG activity, the so-called bereitschaftspotential [8–10] or a change in the proportion of frequency bands in the EEG [11]. Although these events may indicate an increase in cortical excitability [12], this does not seem to go with any increase in corticospinal excitability until the last 50–80 ms preceding movement onset [13]. However, the excitability of subcortical motor structures preceding self-generated tasks has not been investigated. 

Previous research has detailed the differences between processes subserving movements in the context of a reaction time task or as self-initiated movements. Studies performed in monkeys suggested that the medially located supplementary motor area (SMA) is more involved in self-initiated movements and that the lateral premotor area is more related with movements externally triggered [14, 15]. A theory for the generation of brain activity preceding movement onset is an internal timing of action within the motor system, which predicts that reverberant activity in the corticobasal ganglia loop would generate large perimovement discharges and lead to the initiation of action [16]. Recently it has been observed in humans that reactions and self-paced movements can similarly activate the pre-SMA, SMA, and rostral cingulate cortex, but the timing of the hemodynamic response within the pre-SMA was earlier for self-initiated movements [17]. Cortical excitability, measured with the readiness potential (bereitschaftspotential) is greater before self-paced movements than with externally triggered movements [18], and this excitability is thought to arise mainly from the SMA [8]. Functional brain imaging has shown that there is a higher activation of the SMA for self-initiated movements than for externally triggered ones [19, 20]. All these studies suggest that there are some cortical components of voluntary action programming that are prepared earlier than those needed to execute a similar movement in the context of a reaction time task paradigm. However, such cortical activity does not necessarily predict the occurrence of voluntary movement, since it may occur in subjects just imagining the movement [11]. In the process of preparation for task execution, whether self-generated or in the context of a reaction time task, structures and circuits along the motor pathway from cortex to alpha motoneurons that are involved in the execution of the required task have to reach a critical level of excitability enhancement just preceding the release of the motor programme. Such preparation of the subcortical motor structures may be assessed using a startling auditory stimulus (SAS). It is known that, in reaction time task experiments, a SAS delivered at the same time as the imperative signal causes a rapid release of the motor programme, which may be executed by passing the normal cortical execution pathways [21, 22]. A similar effect has been reported in a few studies using anticipation-timing tasks. When the experimental condition allows for subjects to predict the time of imperative signal presentation, preparation becomes delayed with respect to those instances in which there is no anticipatory timing information [23]. However, this may largely depend on the experimental condition, and, in a study devoted specifically to examine the effects of anticipatory timing, Carlsen and MacKinnon [24] found that sufficient preparation has occurred up to 500 ms before the presentation of the cue. Up to date, no study is available on what is the behavior of subcortical motor structures to the presentation of a SAS at the time the subjects are prepared to perform a self-generated action. 

Therefore, we engaged upon a study to determine if SAS was capable of triggering the execution of a voluntary ballistic movement when prepared for execution at free will. We considered this of physiological interest due to the differences reported in the central processes implicated in self-generated and reaction time movements. We also compared the effects of SAS in both conditions, as a surrogate of the degree of preparedness.

## 2. Methods

### 2.1. Ethical Approval

This study was approved by the local Ethics Committee of the Hospital Clinic. All subjects gave their informed consent for the study, which was conducted in accordance with the Declaration of Helsinki. 

### 2.2. Subjects

Eight healthy subjects (5 females and 3 males, aged 28–52) took part in the study. All were self-reported right-handers with normal or corrected-to-normal vision and were free from any neurological deficit that could affect the execution of the task. 

### 2.3. Recording and Stimulation

Subjects were sitting comfortably in a chair. Their upper limb was placed on a home-made metallic structure consisting of two parts, purposefully built to firmly hold the forearm in one and the hand in the other. The distal part, joined with a hinge to the proximal part, could move without resistance in the flexoextension direction. An electrogoniometer (Model X 65; Biometrics; Gwent, UK) attached to the two metallic pieces was used to record the angular position of the wrist joint. Surface electrodes were used to record the electromyographic activity (EMG) of the wrist extensor and wrist flexor muscles through an electromyograph Mystro5Plus (Vickers Medical, Surrey; London), recording mainly from extensor carpi radialis for WE and from flexor carpi ulnaris for WF. The band-pass frequency filter was set at 20–500 Hz for the EMG activity. The signals from the electromyograph and the goniometer output were fed into a computer at a sampling rate of 2000 Hz, for offline analysis with the software Acknowledge, MP100 (Biopac Systems, Bionic, Barcelona). We also recorded the EMG activity from the orbicularis oculi to monitor the startle reaction.

### 2.4. Procedure

Subjects were facing a computer screen positioned at approximately 1 meter distance at eye level. It showed only the goniometer signal trace that, at onset of each trial, started to advance slowly from left to right, according to a screen total time resolution of 6 s. All trials began with a voice forewarning that announced the start of the trial which took place when the experimenter pressed a computer's key after a short random interval. Subjects were requested to perform a ballistic wrist extension under two situations: Reaction and Action. In Reaction, subjects were asked to perform a ballistic wrist extension at the time when the signal from the goniometer reached a vertical black line (the visual imperative signal = IS), which was displayed at a time that randomly varied trial by trial between 3000 and 5000 ms after onset of the recording. In Action, subjects were requested to perform the same movement at any time they wished, between 3000 and 5000 ms after onset of the trace, with no visual cue. To give subjects the opportunity to familiarize themselves with the task they were allowed to practice. After each practice trial they were given feedback on the time of execution of their movement. They were told to avoid counting seconds internally. Test recordings began after practice trials, when subjects managed to perform five consecutive trials within the above mentioned time window. 

In some trials, at random (20–25% of trials), the system was prepared in such a way that there was a trigger out at the time when the goniometer signal reached the visual IS in Reaction trials and at random between the third and the fourth seconds, within the time range of the expected movement in Action trials. The trigger out was used to discharge a magnetic stimulator on top of a metallic platform, which gave rise to a loud auditory stimulus, capable of producing a startle reaction. Such startling auditory stimulus (SAS) has been measured to be of an intensity of 130 dB sound pressure level (SPL), when delivered at a distance of 1 m from the source [21]. For each subject the study procedure was completed in two blocks. The Reaction block contained 24 trials, divided into 20 reaction time trials (RT) and, randomly interspersed among them, 4 trials in which SAS was applied together with the IS (RS). The Action block contained 60 trials, 48 action time trials (AT) and, randomly interspersed among them, 12 trials containing SAS (AS), which we attempted to apply at a time that could coincide with motor preparation for free will execution of the task. In order to do that, we considered a time span sufficiently wide for the subjects to act with free will and, at the same time, sufficiently narrow to allow for SAS to precede the voluntary action in a few chance trials. Since there is a known time window of efficacy for SAS to induce the StartReact effect in reaction time tasks [7, 25], we concentrated SAS in AS trials during the middle part of the time window allowed for task execution and hence avoided applying SAS during the first 400 ms and during the last 1000 ms. Consequently, we distributed the 12 SAS at every 50 ms within the 600 ms epoch ranging from 3400 to 4000 ms after onset of the trial, hoping that there would be one or two SAS per subject coinciding with the expected preparation before execution. The entire Action block was repeated in a second session (another block of 60 trials) in 2 subjects who had no trial in which SAS preceded task execution. 

Before the experiment, we delivered a control trial with SAS only, to make sure that subjects were actually responders to SAS. To reduce fatigue and keep the attention, subjects had a first 5–10 min break between Reaction and Action blocks, and a second 5–10 min break halfway through the Action block. 

### 2.5. Data Analysis

We considered three types of AS trials, according to the possible distribution of subject's actions around SAS. (1) Action execution occurred before SAS was delivered (ASb), (2) Action execution occurred long late after SAS was delivered (ASl), and (3) SAS was delivered close before action execution (ASc). We judged that only ASc trials would have the possibility to produce an effect on the intended action. We grouped the results obtained in each subject according to one of the five conditions: RT, RS, ASb, ASc, and ASl. The primary outcome measure for RT and RS trials was premotor reaction time, defined as the time between IS and onset of extensor muscles EMG activity. For ASb, ASc, and ASl trials, we determined the “SAS-to-action” time, defined as the time between the SAS delivery and extensor muscles EMG onset (either positive or negative according to whether SAS was applied before or after action onset). For the orbicularis oculi (OOc) we measured time onset in a time window after IS, ranging from 30 to 140 ms. Both EMG and movement recordings were considered at the first deviation from baseline that was larger than 50 *µ*Vs for a duration of at least 50 ms. In rectified EMG activity, we calculated the area under the curve (iEMG) for the first 50 ms following extensor muscles EMG onset for each subject and condition and for OOc when SAS was applied. Data were normalized and expressed as percentage of the mean value at RT condition. For OOc measurements data values of the RS condition were considered as reference for normalization.

Movement latency was measured at movement onset in the signal from the goniometer, from the IS in RT and RS trials and from SAS in AS trials being reported as mean and standard deviation. For ASb, ASc, and ASl trials we determined the time at which the voluntary wrist extensor EMG activity began with respect to the onset of the trial. In a separate descriptive analysis, we determined the time at which movement onset began in AT trials. These time points, freely chosen by subjects, giving a random and scattered distribution of data, were reported as median, minimum, maximum, and quartile values. We calculated movement time as the time elapsed from movement onset to movement end, peak velocity, as the maximum peak of the first derivative of displacement over time, and time to peak velocity. In RS and ASc trials we also measured the latency of the response from the OOc after SAS. 

The primary outcome measure for ASb, ASc, and ASl trials was the distribution of trials around SAS. We considered that, if there was no effect of SAS on the subject's voluntary action, onset latency of EMG activity and movement signal would have a scattered and even distribution around SAS. However if SAS had an effect, we expected that ASc trials would gather as a cluster, separated by a relative gap from all ASb and ASl trials. 

We compared data from the same subjects in different conditions using one-factor ANOVA for group comparisons. Levene's test for homogeneity of variance was used. Post hoc comparisons were done with the Bonferroni correction when the main effect of condition was significant. For post hoc comparisons Scheffe or Games-Howell tests were used when appropriate. Level of significance was established at *P* = 0.05 or corrected according to total number of comparisons. Standardized effect sizes are provided as partial eta squared values (*η*_*p*_^2^). For graphic representation, we chose to show data as mean ± 1 SD.

## 3. Results

**Figure 1:**
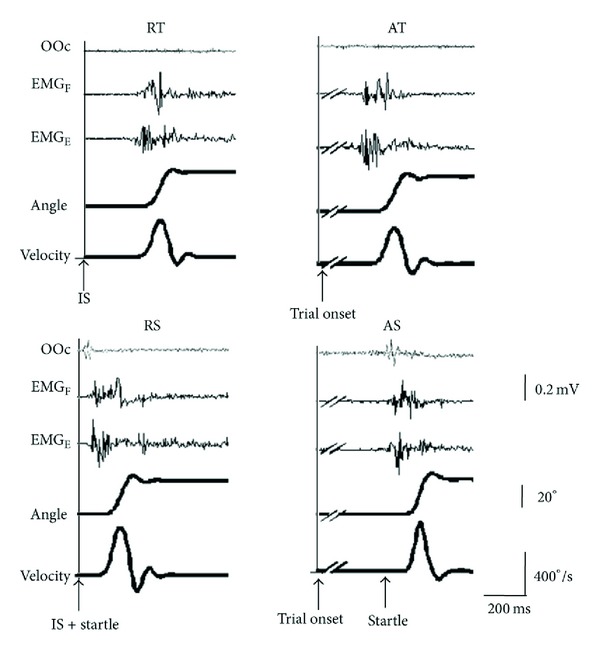
Representative trials from a single subject. RT: trial obtained in response to an imperative signal (IS). RS: trial obtained in response to an imperative signal, delivered together with a startling auditory stimulus (SAS). AT: trial obtained in a self-initiated action performed within the required time window. AS: trial obtained in a self-initiated action performed within the required time window short time after a SAS. OOc: EMG activity recorded from the orbicularis oculi muscle. EMG_F_: EMG from wrist flexor muscles. EMG_E_: EMG from wrist extensor muscles. Wrist extension is shown as an upwards change in the angle and velocity traces.

Subjects performed the movement resulting in an overall mean wrist extension angular displacement of 54.56 ± 10 degrees without significant differences among conditions (*F*_4,35_ = 0,43, *P* = 0.784, *η*_*p*_^2^ = 0.047).  Figure 1 displays representative selected trials from a subject for each condition, and Table 1 includes mean data for each condition and all variables. In the AT block, subjects performed the fast movement by their own will within the set time range in 95% of the trials. Trials that were out of the required time window (24 trials, 5%, 2 with SAS) were not considered for subsequent analysis. 

**Figure 2:**
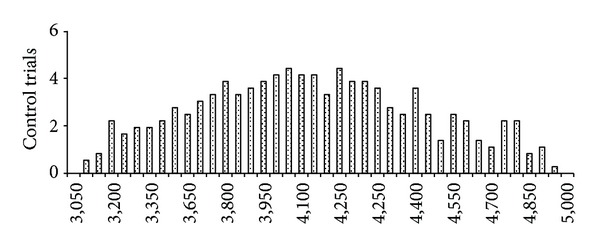
Histogram of the temporal distribution of EMG latency for AT trials in bins of 50 ms along the epoch of interest.

**Figure 3:**
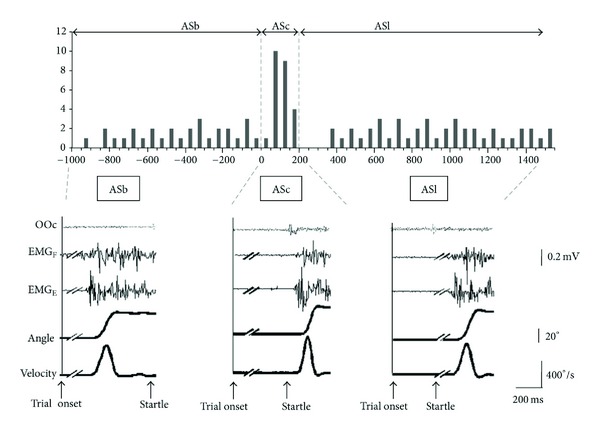
Upper part: histogram of the temporal distribution of all EMG latencies for AS trials in bins of 50 ms normalized with respect to the time of startling auditory stimulus (SAS) application, at time 0. Lower part: representative trials from a single subject. ASb: trial before startle. ASc: trial close after SAS. ASl: trial late after startle. OOc: EMG activity recorded from the orbicularis oculi muscle. EMG_F_: EMG from wrist flexors. EMG_E_: EMG from wrist extensors. Wrist extension is shown as an upwards change in the angle and velocity traces.

The temporal distribution of trials in which subjects performed the movement without visual cue (AT trials) is shown in bins of 50 ms in Figure 2. The median EMG latency time elapsed since trial onset was 3.92 ± 0.15 s (CI 95% for the first quartile: 3.63 ± 0.19 s and CI 95% for the third quartile: 4.25 ± 0.21 s). The analysis of the effects of SAS includes data from 94 AS trials since 2 trials were discarded due to the fact that the movement was performed out of the set time window. The “SAS-to-action” time, normalized with respect to the time point of SAS application (considered 0), is plotted in Figure 3 for each of the 94 trials in bins of 50 ms. This figure shows that extensor muscle EMG activity began before SAS delivery (ASb) in 29 trials, which were evenly distributed in the time domain. The distribution of the 66 remaining trials was not uniform. Instead, in 24 trials (ASc) movement onset occurred within 200 ms after SAS, followed by a gap of about 200 ms, and in the remaining 41 trials (ASl) movement onset was again evenly distributed beyond that time point.

For RT, RS, and ASc conditions differences were observed in latency of EMG (*F*_2,21_ = 105,8, *P* < 0.001, *η*_*p*_^2^ = 0.910) and latency of movement onset (*F*_2,21_ = 76,45, *P* < 0.001, *η*_*p*_^2^ = 0.879). Concerning variables related to movement performance there were also differences among all five conditions: movement time (*F*_4,35_ = 12,46, *P* < 0.001, *η*_*p*_^2^ = 0.587), time to peak velocity (*F*_4,35_ = 17,14, *P* < 0.001, *η*_*p*_^2^ = 0.662), peak velocity (*F*_4,35_ = 23,98, *P* < 0.001, *η*_*p*_^2^ = 0.733), and iEMG (*F*_4,35_ = 18,35, *P* < 0.001, *η*_*p*_^2^ = 0.677). These comparisons are described into more detail in the subsequent paragraphs.

In trials containing SAS, the OOc showed an EMG burst that was similar in all conditions (see Table 1) without significant differences concerning latency (*F*_3,28_ = 2,41, *P* = 0.09, *η*_*p*_^2^ = 0.29) or size (*F*_4,35_ = 1,59, *P* = 0.2, *η*_*p*_^2^ = 0.146).

### 3.1. Effects of SAS on Reaction

The analysis of trials in which subjects had to react to the visual cue showed the expected shortening of reaction time, latency of movement, and movement time when the SAS was applied in RS trials, with respect to the RT trials (Table 1). These differences as well as an increased agonist EMG burst were significant. The analysis also showed higher peak velocity (see Table 2). 

### 3.2. Effects of SAS on Action

**Figure 4:**
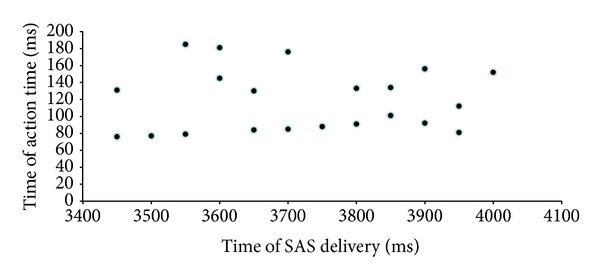
Distribution of all EMG latencies for ASc trials along the whole epoch in which the SAS was applied. Temporal relationship between time of SAS application across the time window and latency of Action close to SAS (ASc) trials from SAS.

The ASc trials formed a narrow cluster of 21 trials with a mean latency of 104.71 ± 17 ms after SAS delivery, while the rest of trials (ASb and ASl) had a broad scattered distribution. All subjects gave ASc, ASb, and ASl responses. We ruled out the possibility of expectancy by showing that the ASc trials were distributed along the whole predetermined epoch for SAS delivery, with no higher probability of occurrence at any of the bins (Pearson's Coefficient = 0.04; *P* > 0.05) (Figure 4). EMG and kinematic data are shown in Table 1. Post hoc ANOVA, comparing ASc with ASb and ASl showed no significant differences for the angular displacement but significant differences for other characteristics including shorter movement time, shorter time to peak velocity, and higher peak velocity (Table 2). The amount of initial agonist activity (iEMG) was larger for ASc although not reaching significance. 

### 3.3. Similarities in the Influence of SAS on Reaction and Action Movement Preparation and Execution

To assess whether there were specific differences between RS and ASc with regard to programme preparation and execution, both conditions were compared for EMG activity and kinematic variables. Latency with respect to SAS, the primary outcome measure, was not significantly different among conditions, and there were no other significant differences in the remaining EMG or kinematic variables (Table 2).

### 3.4. Similarities in Preparedness on Reaction and Action Movements Execution

To compare to what level preparedness lead to similarities between these two ballistic scenarios (reaction and action at will) RT was compared with ASb and ASl. As can be seen in Table 2, in the RT condition no significantly shorter movement time, reduced time to peak velocity, higher peak velocity, nor higher muscular activity was observed during the first 50 ms (iEMG) when compared with the ballistic actions performed at will (ASb and ASl). 

## 4. Discussion

This study shows that a loud auditory stimulus influences the execution of self-generated human actions in a way similar to what is known to occur in simple reaction time trials. With this study we have further explored the relationship between prepared motor actions and the startle pathways. The speeding-up effect of a SAS on the execution of internally generated motor acts has not been investigated before. The effect does not seem to be stochastic in nature but rather depends on the coincidence between SAS and the assumed building up of preparatory activity just before execution of the willed motor action [26]. In our study, SAS had an effect in some trials and not in others, with a clear separation among them. Our interpretation of such observations is that in some trials the SAS was able to access the motor structures prepared for execution of the motor programme whereas the effects were not observed when those structures were not highly prepared. One collateral aspect issuing from our findings is that they rule out intersensory facilitation as an explanation for the effects of a SAS on movement execution [27]. No external commands were used to trigger AT or AS, and, therefore, there are no grounds for intersensory facilitation. 

Although in first instance a similar process in the preparation of reactions and internally driven actions may be expected, there are significant differences between them: there are different cerebral areas activated for each process [14, 15, 19, 20], a different buildup in time of cerebral potentials [18], and different response characteristics [28–30]. In both conditions, however, SAS speeded up the execution of the prepared motor programme with no evident changes in its structure, an effect shown previously by several authors [21, 22, 31–34], suggesting that the effect of SAS is integrated in the ongoing task. 

Up to now, most of the research on the so called StartReact effect has dealt with reaction time tasks, where the temporal aspects of the reaction can be experimentally controlled. Some authors, though, have investigated how the preparation to perform an internally generated action modified the execution of the same or another action in a reaction time task [28, 35]. They showed that, when subjects had to react during the building up of an internally generated preparation to act, the reaction time responses were delayed in comparison to the same reaction done without an ongoing internal preparation. The delay was different according to whether the responses were congruent or not; that is, the delay of the reaction time response was less prominent when the responses required were the same for both (action and reaction) compared to when the response required for the reaction time task was different than the one being built internally. The authors concluded that subjects could not take advantage of the ongoing preparation of internally generated actions to perform externally driven reactions and, therefore, they suggested that the two circuits are clearly separated. It is to note, though, that the main source of delay in reaction time in these conditions might have been in the processing of the afferent signal rather than in the execution since the same delay was observed in simple and complex reaction time tasks. Using a similar paradigm Hughes et al. [36] also found that time of preparation mattered: when subjects were in a more advanced preparation for voluntary action their response in a congruent reaction was indeed faster than with less advanced preparation. The authors concluded that the stimulus-driven system could take advantage of the high degree of preparation of the voluntary system, and thus both systems might share common central preparatory mechanisms. In an attempt to reconcile these two opposite views, we should consider what may account for the differences in processing and similarities in execution. Likely, the processing time for an afferent input to reach the motor execution areas may take longer when our sensorimotor areas are engaged in an internally generated buildup of excitability since this may block externally generated sensory inputs that would potentially interfere with such process [37], thus likely accounting for the reported delay attributed to the afferent circuit. However, when subjects are highly prepared, the amount of excitability builtup in the execution system may change the situation. Beyond a certain point in preparation, everything in the motor system may be ready to trigger the prepared motor programme. In our opinion, this high degree of readiness in the motor system is what actually leads to the StartReact phenomenon and may also explain the faster execution of the congruent response found by Hughes et al. [36], since at that point low intensity stimuli may behave as a startle-like inputs [26]. 

Our findings in the present study are in agreement with the considerations made in the previous paragraph. We interpret that the cluster of actions with a latency within the first 200 ms from the application of SAS (ASc) was made up by those trials in which the preparation to perform the internally generated action reached beyond a certain level, at which the SAS could trigger the execution of the motor programme. In our subjects, latency of SAS-induced action execution (in ASc trials) was not different from latency of the SAS-induced reaction execution (in RS trials). Therefore we believe that there was no interference between both systems. Probably subjects did not shift from an internally driven command to an externally driven command but just anticipated the execution of the prepared movement when they reached an adequate level of excitability enhancement in their motor pathway. The observations reported by Kumru et al. [38] support the idea that the intensity of the StartReact effect depends on the degree of excitability of the motor system in preparation to act. In this study subjects showed faster execution in reaction time experiments involving forced choice than in those involving a Go/noGo condition. It has also been demonstrated that just the desire to act leads to a state of high excitability [39, 40]. Accordingly, preparation for a motor act requires building up the programme with an increase in excitability in all structures that participate in its execution along the motor pathway.

Not only response latency but also other characteristics of the resulting movement were similar for ASc and RS trials. Therefore, our results suggest that the mechanisms engaged in movement preparation for volitional execution share some similarities with those engaged in responding to an external cue. Notwithstanding, cortical preparation is different for both tasks: long preparation in internally generated commands and just a premotor potential in movement execution in a reaction time paradigm. Motor preparatory activity has been described in brain areas up to 2 seconds before voluntary actions [8, 41], and, in case of choice actions, prefrontal cortex activity occurs several seconds before action [42]. The Bereitschaftpotential, a slowly rising negative action potential recorded from the scalp, appears in movements made with free will [8, 43, 44] and may indicate some long lasting preparation that begins well in advance of the generation of descending motor commands. However, the time window in which the SAS effectively triggered the action was limited to 300 ms. A somewhat longer time window, of about 500 ms of sustained excitability enhancement before the imperative signal, was suggested by Valls-Solé [25] in a simple reaction time task paradigm. The difference may be due to raised expectation of the cue after forewarning in reaction time tasks. 

Although there are limitations in free will studies because of the implicit difficulties in introducing temporal experimental constraints, we believe that a motor act can be considered an act of free will when subjects have a certain freedom [45] such as decision on the time to act, as they had in our experiment. Our subjects were free to choose the time point of action, and, given the even distribution of the AT trials within the 2-second period allowed, we can assume that they did so. However, even if actions were performed at will, our subjects responded to the SAS as an external stimulus, suggesting that the effect of SAS on action occurred at a similar site as that on reaction. A comparative study between reaction, and action was done by Cunnington et al. [17] using event-related fMRI. These authors explored subjects performing finger tapping as a reaction to a visual stimulus or at their own will. For both conditions similar activations at medial motor and selected cortical areas were observed. However, activation within the basal ganglia was found only for self-generated movements. Their subjects showed a similar level of supplementary motor area (SMA) and cingulate cortex activation for both movement conditions, but at pre-SMA the activation timing was earlier for self-initiated movements (1.48 s of difference). These findings indicate that pre-SMA and basal ganglia play a relevant role in differentiating internally generated from externally triggered actions [45]. In our study, the fact that responses in reaction time tasks were similar in general shape to those shown by actions performed close after SAS delivery, supports the idea that the influence of SAS in both, action and reaction, has to occur at a level where the two types of tasks coincide, out of cortical and basal ganglia processing. We consider that a potential site along the motor pathway for the SAS to release the prepared motor programme is located at subcortical motor centers with enough hierarchy to activate a sufficient number of motoneurons for full motor programme execution. Previous research has suggested that this formation is likely the brainstem reticular formation [21, 22, 26, 33]. However, in an ingenious recent work, Alibiglou and MacKinnon [46] have advocated that the effects of a startle on movement execution require a cortical transit. Our results indicate that, if this is the case, such cortical structure should have reached a similar level of preparation in both conditions at a certain point to be ready to release the motor programme after SAS delivery.

Our study has some limitations. We did not stress accuracy in the kinematics of movement performance but just requested a quick initial reaction. We considered that latency would be a more reliable measure than movement kinematics in terms of comparison with previous studies concerning movement preparedness. Therefore, we do not know if some differences would exist in the characteristics of the movement between internally generated actions and externally triggered reactions if we had stressed accuracy in movement performance. We cannot completely exclude a carry-over effect between blocks, although they were presented with a time separation of 30 minutes which might have been enough to prevent significant effects. The fact that no changes were observed in the size of the OOc response as an index of the effect of the startling stimulus in trials of interest supports this. Finally, our reaction time task was actually an anticipation timing task, as subjects were familiarized with the time frame in which the response had to be performed. However, it has been shown that this type of reaction time task responds to the presentation of a SAS in a similar way as simple reaction time tasks, except for some modulation according to the level of information given [23, 24].

Our results suggest that, whether subjects prepare their motor system to respond to an external cue or to perform a willed movement, they do so by enhancing the excitability of structures of the motor pathway at subcortical level, where they are accessible to a SAS. The release of the motor programme by SAS does not require the combination of two stimuli, and, therefore, it does not seem to involve reinforcement of afferent input but rather a timely occurrence in the preparation phase of movement execution. 

## 5. Conclusions

A startling auditory stimulus anticipates the execution of self-generated human actions in the same way as it shortens the latency of task execution in simple reaction time trials. 

The anticipation may depend on the coincidence between SAS and the assumed buildup of preparatory activity just before execution of the willed motor action. 

The programme for willed execution of fast actions may entail the preparation of subcortical motor structures that can be accessed by a startling stimulus to trigger the response.

## Supplementary Material

Table 1: EMG and kinematic values for trials in which subjects performed a fast reaction with a wrist extension or a fast wrist extension at will.
Table 2: Post-hoc analysis for selected comparisons of interest.
Fig. 1. Representative trials from a single subject for all conditions.
Fig. 2. Histogram of the temporal distribution of EMG latency for Action-time trials.
Fig. 3. Histogram of the temporal distribution of all EMG latencies for Action-SAS trials, and representative trials for the three types of Action-SAS trials.
Fig. 4. Distribution of all EMG latencies for those Action-SAS trials in which SAS was applied close before action execution.

## Figures and Tables

**Table 1:** EMG and kinematic values for trials in which subjects performed a fast reaction with a wrist extension or a fast wrist extension at will.

Measures	RT	RS	ASb	ASc	ASl
Angular displacement (°)	55.04 ± 6	53.63 ± 11	52.33 ± 7	51.04 ± 14	56.11 ± 14
Latency EMG (ms)	235.9 ± 35	89.1 ± 20		88.13 ± 17	
Latency of movement (ms)	287.4 ± 37	146.7 ± 9		145.88 ± 24	
Movement time (ms)	156.4 ± 17	127.9 ± 9	149.1 ± 8	123.75 ± 10	151.25 ± 11
Time to peak velocity (ms)	85.23 ± 13	57.6 ± 12	96.25 ± 13	58.25 ± 10	99.38 ± 19
Peak velocity (°/s)	414.4 ± 43	577.0 ± 60	409.88 ± 49	589.01 ± 66	393.38 ± 59
iEMG*	100	318.1 ± 101	104.05 ± 32	236.38 ± 93	107.88 ± 20
OOc EMG latency		46.68 ± 4	46.50 ± 4	49.38 ± 4	50.63 ± 3
OOc EMG size		8225.5 ± 2244	6379.88 ± 1768	6186.76 ± 2209	6729.1 ± 2015

RT: values for the control trials in response to an imperative signal (IS). RS: values for the experimental trials in response to the IS with simultaneous startle. ASc, ASb, and ASl are wrist extension at will performed, respectively, close after SAS, before SAS, and long after SAS. *Reference values are 100 (dimensionless).

**Table 2:** Post hoc analysis for selected comparisons of interest.

Measures	RT versus RS	ASc versus ASb	ASc versus ASl	ASc versus RS	RT versus ASb	RT versus ASl
Angular displacement (°)**, a	0.915	0.999	0.953	0.853	0.993	0.993
Latency EMG (ms)**, b	***0.000***	n.a.	n.a.	0.989	n.a.	n.a.
Latency of movement (ms)**, b	***0.000***	n.a.	n.a.	0.995	n.a.	n.a.
Movement time (ms)*, a	***0.001***	***0.004***	***0.002***	0.974	0.816	0.943
Time to peak velocity (ms)*, a	0.009	***0.000***	***0.000***	1.000	0.642	0.399
Peak velocity (°/s)*, a	***0.000***	***0.000***	***0.000***	0.996	1.000	0.967
iEMG first 50 ms**, a	***0.003***	0.028	0.040	0.475	0.996	0.863

n.s.: no significant differences; n.a.: nonapplicable; Scheffe test*; Games-Howell test**; a: significant level at 0.005; b: significant level at 0.017; significant differences are in bold and italics.
