# Supplementary material for: The StartReact Effect on Self-Initiated Movements
Source: Biomed Res Int. 2013 Sep 11;2013:471792. doi: 10.1155/2013/471792 (PMC3784278; doi:10.1155/2013/471792)
Supplement: Supplementary file 1 — Table 1: EMG and kinematic values for trials in which subjects performed a fast reaction with a wrist extension or a fast wrist extension at will. Table 2: Post-hoc analysis for selected comparisons of interest. Fig. 1. Representative trials from a single subject for all conditions. Fig. 2. Histogram of the temporal distribution of EMG latency for Action-time trials. Fig. 3. Histogram of the temporal distribution of all EMG latencies for Action-SAS trials, and representative trials for the three types of Action-SAS trials. Fig. 4. Distribution of all EMG latencies for those Action-SAS trials in which SAS was applied close before action execution. [file 471792.f1.pdf]

PHYSICAL MEDICINE AND REHABILITATION DEPARTMENT
FACULTY OF MEDICINE
UNIVERSIDAD COMPLUTENSE DE MADRID

C/Ciudad Universitaria s/n
28040 Madrid
Spain

E-mail: jmcastel@med.ucm.es

16 July 2013

Study Title: **The StartReact effect on self-initiated movements**
Ref: 2738/471792.v1

Dear Editor in Chief,

Thank you for your letter regarding the article of reference submitted for evaluation for publication in BioMed Research International. We have carefully read the comments of the reviewers, and decided to provide the following answers. We have modified all sections of the manuscript according to the suggestions, as shown in the attached version.

We hope that the changes made in the manuscript satisfy the concerns of the reviewers and the article is now acceptable for publication.

Yours sincerely,

Juan M Castellote
